# Distinct actions of the humid heat environment on host gut microbiota, intestinal mucosal immunity, neuroendocrinology in influenza A virus-infected mouse

**DOI:** 10.1016/j.bbih.2025.101164

**Published:** 2025-12-19

**Authors:** Sizhi Wu, Yiwen Lv, Peng Pang, Huachong Xu, Li Deng, Wei Ma, Xiaoyin Chen

**Affiliations:** a Department of Geriatrics, Guangzhou First People's Hospital, South China University of Technology, Guangzhou, China; b College of Traditional Chinese Medicine, Jinan University, Guangzhou, China; c The Second Affiliated Hospital, Guangzhou Medical University, Guangzhou, China

**Keywords:** Humid heat environment, Influenza A virus, Gut microbiota, Gut-brain axis, Metabolomics

## Abstract

**Background:**

Climate factors exert a profound influence on human emotional well-being and physical health. Exposure to a humid heat environment is known to precipitate anxiety-like behaviors and exacerbate the clinical manifestations of influenza; concurrently, mounting evidence has demonstrated a bidirectional regulation between the gut microbiota and human health, suggesting a potential link between environmental stress and microbial homeostasis.

**Methods:**

In this study, C57BL/6J male mice were subjected to a humid heat environment for 3 weeks prior to infection with the influenza A virus. Microbiota composition, metabolites, and intestinal mucosal immunity were comprehensively measured. Furthermore, behavioral phenotypes and neurotransmitter levels were assessed to explore their potential correlations with gut dysbiosis.

**Results:**

Exposure to a humid heat environment aggravated pulmonary and intestinal tissue damage while reshaping the gut microbiota composition and metabolome. This environmental stress precipitated severe pathological injury and robust inflammatory in the intestinal mucosa, characterized by a multifold upregulation of Th1/Th2-related cytokines and the suppressed expression of *Ocln*, *ZO-1*, *pIgR*, and *SIgA*. Further experiments revealed that the humid heat environment exacerbated neurological deficits in influenza A virus-infected mice, accompanied by a significant reduction in neurotransmitter levels. **Conclusions**: These data demonstrate that exposure to a humid heat environment exacerbates influenza infection severity through the dysregulation of the intestinal homeostasis and the neuroendocrine system, revealing the potential mechanisms underlying the digestive and nervous system symptoms observed in influenza patients.

## Highlights

• Exposure to a humid heat environment exacerbated gut microecological dysbiosis in IAV-infected mice.
• Humid-heat exposure resulted in more severe intestinal inflammatory responses.
• Reduced key neurotransmitters were observed in IAV-infected mice under humid heat conditions.
• These changes were associated with aggravated anxiety- and depression-like behaviors in the IAV mouse model.

## Graphical abstract

**Figure:**
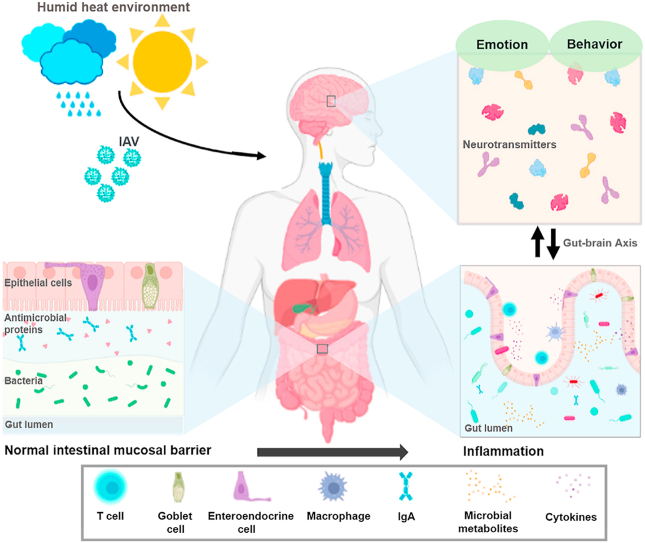

## 1 Introduction

Environmental factor, including humidity, temperature and light, are closely related to public health. Influenza is an epidemic acute respiratory disease worldwide, characterized by high morbidity and mortality. Influenza epidemics show distinct summer and winter peaks, indicating the significant role of climatic conditions in disease pathogenesis (Chong et al., 2020). In addition to fever and cough, influenza is often accompanied by gastrointestinal and neurological symptoms, such as abdominal pain, diarrhea, lethargy, and drowsiness (Okayama et al., 2011; Ozdemir et al., 2011; Vivar and Uyeki, 2014). There is a clear correlation between the incidence of influenza and climate changes (Zheng et al., 2021). Therefore, it is of great value to investigate the mechanisms by which climatic conditions might affect the onset and progression of influenza.

Mounting evidence suggests that there is bidirectional communication between the lung and gut microbiota (Bhattacharya et al., 2022; Dessein et al., 2020; Groves et al., 2018; Marrella et al., 2024). In addition, the gut microbiota plays an important role in neuroendocrine function, altering gene expression in critical brain regions and leading to behavioral perturbations in mice (Burokas et al., 2017b). These effects have been demonstrated in experiments with *Bifidobacteria* and *Lactobacilli*, members of the gut microbiota that have been utilized to improve brain health (Allen et al., 2016; Savignac et al., 2015). Furthermore, studies have indicated that exposure to humid heat has a significant impact on human physiological and perceptual responses (Xu et al., 2025), and can also regulate human sleep stages and body temperature (Okamoto-Mizuno et al., 2005). Therefore, we hypothesize that climate effects on influenza may be mediated by regulating the gut microbiota, gut-brain axis, and neuroendocrine system.

In previous studies, we showed that the living environment, specifically a humid heat environment, also significantly affects the composition of the gut microbiota (Deng et al., 2020) and may contribute to influenza pandemics through immune suppression (Wu et al., 2015). Moreover, a humid heat environment could induce anxiety-like disorder by regulating the gut microbiota and bile acid metabolism in mice (Weng et al., 2024). Gene-environment interactions, as well as gut-brain regulation, may play a key role in the development of irritable bowel syndrome (Raskov et al., 2016). Notably the gut is closely connected to the central nervous system through dynamic bidirectional communication along the gut-brain axis, which may further influence the mood and behavior of the host (Moran and Thapaliya, 2021). However, the effects of a humid heat environment on the intestinal metabolite composition and neuroendocrine system of influenza A virus (IAV)-infected mice remain unclear, warranting further investigation.

In this study, we investigated whether administration of a humid heat environment affects IAV infection, concomitant with associated changes in gut microbiota composition and metabolite production. We also assessed the impact of a humid heat environment in combination with IAV infection on behavior and the neuroendocrine system.

## 2 Methods and materials

### 2.1 Animals

In this study, male C57BL/6J mice (*n* = 48, five-week-old; Laboratory Animal Center of Guangdong Province, China) were utilized. All experiments were conducted in strict accordance with the Guide for the Care and Use of Laboratory Animals (ninth edition; National Institutes of Health, Bethesda, MD, USA) and were approved by the Animal Ethics Committee of Jinan University (Approval No. 20160616103002.).

### 2.2 Ambient exposure

**Fig. 1:**
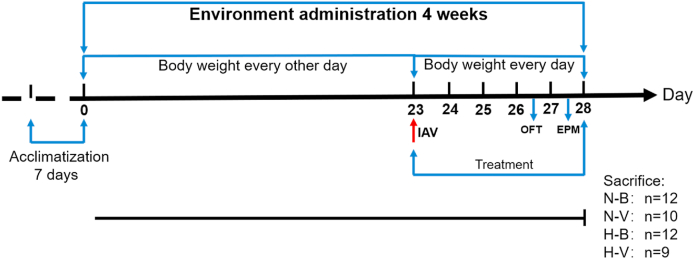
Experimental design. Animals underwent a four-week experimental period. Mice in the N-B and N-V groups were housed in a normal environment, while mice in the H-B and H-V groups were maintained in a humid heat environment for 28 days. On day 23, mice in the N-V and H-V groups were intranasally administered diluted IAV, while the other mice received saline for comparison. Behavioral tests were conducted as follows: day 26, open-field test (OFT); day 27, elevated plus maze (EPM). Mortality was observed post-infection: in the N-V group, 2 mice died between day 24 and day 27; in the H-V group, 3 mice died during the same period. At the end of the experiment, all mice were blood-sampled via retro-orbital bleeding and immediately euthanized by cervical dislocation. Subsequently, target tissue samples were collected. Group abbreviations are as follows: N-B, normal environment control; N-V, normal environment with IAV infection; H-B, humid heat environment exposure; H-V, humid heat environment exposure with IAV infection.

Following a one-week acclimatization period in a specific pathogen-free (SPF) facility under controlled conditions (22 ± 1 °C, 55 % ± 5 % relative humidity, 12-h light/dark cycle), 48 six-week-old mice (19.78 ± 0.9152g) were randomly assigned to the following four groups **(**n = 12 per group**)**: normal environment control (N-B), normal environment with IAV infection (N-V), humid heat environment control (H-B), and humid heat environment with IAV infection (H-V). Mice in the N-B and N-V groups were housed under standard conditions (22 ± 1 °C, 55 % ± 5 % relative humidity), while mice in the H-B and H-V groups were maintained in a humid heat environment (32 ± 1 °C, 95 % ± 5 % relative humidity) for 28 days. All animals were kept on a 12-h light/dark cycle. (Fig. 1).

### 2.3 IAV infection

The mouse-adapted influenza A virus strain A/FM1/1/47 (H1N1) was provided by the Department of Pathogenic Microbiology and Immunology and propagated in the College of Traditional Chinese Medicine at Jinan University. After 22 days of exposure to their respective environments, mice in the N-V and H-V groups were intranasally inoculated with diluted virus (1:80, 50 μL, 20 % LD50), based on our previous experimental data (Deng et al., 2020), under light anesthesia (4 % isoflurane for 5 s). Mice in the control groups were mock-infected with 50 μL of saline.

### 2.4 Histopathology examination

Lung and intestinal samples fixed in 4 % paraformaldehyde were embedded in paraffin, sectioned (4–5 μm thickness), deparaffinized, rehydrated, and stained with hematoxylin and eosin **(**H&E**)**. Histopathological changes were observed under a microscope and quantified using Image-Pro Plus software (Media Cybernetics, Rockville, MD, USA). Lung pathology was scored based on epithelial tissue damage, hemorrhagic congestion, mucosal edema, and neutrophil infiltration (see Supplement 1.2). For colon histopathologic scoring, the evaluated parameters were mucosal thickening, goblet cell depletion, cellular infiltration, and tissue damage. In addition, the length and number of villi were included in the small intestinal histological score. Each parameter was scored on a scale from 0 (normal) to 3 (severe damage) (Bhattacharya et al., 2022). The overall histological score was calculated as the sum of the scores of individual parameters.

### 2.5 Quantitative real-time polymerase chain reaction (qRT‒PCR)

Total RNA was extracted using the PrimeScriptTM RT Reagent Kit (TaKaRa, Japan) and treated with DNase per the protocol (see Supplement 1.3), and all qRT-PCR primers are presented in Table S1.

### 2.6 Enzyme linked immunosorbent assay (Elisa)

Levels of immunoglobulin M **(**IgM) in plasma, IgA and secretory immunoglobulin A (SIgA, the IgA that is secreted into the intestinal cavity) in the ileum were detected using ELISA kits (Multisciences, China; see Supplement 1.4). The reagents and samples were prepared according to the instructions, and the concentration was detected and calculated using a multifunctional enzyme marker (Thermo Fisher, Varioskan LUX, USA).

### 2.7 Cytokine quantification using bio-plex multiplex assay

Cytokine levels were measured using the Bio-Plex Pro™ Mouse Cytokine Th1/Th2 Assay Kit (Bio-Rad, USA). The specimen pretreatment and experimental procedures were performed according to the instructions (see Supplement 1.5).

### 2.8 16S rRNA gene sequencing and analysis

Total genomic DNA was extracted from cecal contents using the GenElute™ Fecal DNA Isolation Kit (Sigma-Aldrich, Germany). DNA concentration and purity were monitored on 1 % agarose gels. The hypervariable V3-V4 region of the bacterial 16S rRNA gene was amplified using Phusion® High-Fidelity PCR Master Mix with GC Buffer (New England Biolabs). The primers used were 338F (5′-ACTCCTACGGGAGGCAGCAG-3′) and 806R (5′-GGACTACHVGGGTWTCTAAT-3′). Data processing and bioinformatics analysis were performed as previously described (Caporaso et al., 2010; Haas et al., 2011; Rognes et al., 2016) (see Supplement 1.6).

### 2.9 Behavior tests associated with influenza symptoms

The open field test (OFT) and elevated plus maze (EPM) were used to assess the behavior of mice as previously described (Burokas et al., 2017a) and detailed in the Supplement (see Supplement 1.7 and 1.8).

### 2.10 Ultra-performance liquid chromatography/tandem mass spectrometry (UPLC-MS/MS) analysis

Cecal content samples were preprocessed following standard protocols (Metware Biotechnology). The extracts were analyzed using an LC-ESI-MS/MS system (UPLC, ExionLC™ AD). Multivariate statistical analysis of differential metabolites among the four groups was performed using supervised principal component analysis (PCA) hierarchical clustering, and orthogonal partial least squares-discriminant analysis (OPLS-DA). Functional annotation and pathway enrichment analysis were conducted using the Kyoto Encyclopedia of Genes and Genomes (KEGG) database**.** Data analysis was performed using R software (version 3.5.0), the ComplexHeatmap package, and MetaboAnalystR. (see Supplement 1.9).

### 2.11 Quantification of short-chain fatty acids (SCFAs)

SCFAs were extracted from fecal samples using methyl tert-butyl ether (MTBE) and quantified by gas chromatography-tandem mass spectrometry (GC-MS/MS) using an Agilent 7890B system coupled to a 7000D mass spectrometer (Agilent Technologies, USA). The analysis was performed in multiple reaction monitoring (MRM) mode. Detailed procedures regarding sample preparation, extraction, and instrumental conditions are provided in the Supplementary Materials. (see Supplement 1.10).

### 2.12 Statistical analysis

Data analysis was performed using SPSS software, version 22 (IBM Corp., Armonk, NY, USA). Bacterial compositional and behavioral nonparametric data were analyzed using the Kruskal-Wallis test followed by Dunn's post hoc test. For all other data with normal distribution, one-way ANOVA was conducted, followed by Bonferroni post hoc test. Correlation analyses were performed using Spearman's correlation coefficient. Statistical significance was set at *P* < 0.05. GraphPad Prism 9.0 was used for data visualization, and Adobe Photoshop CS6 was used for figure assembly. Biorender was used for Graphical Abstract.

## 3 Results

### 3.1 Effects of exposure to a humid heat environment on lung infection

**Fig. 2:**
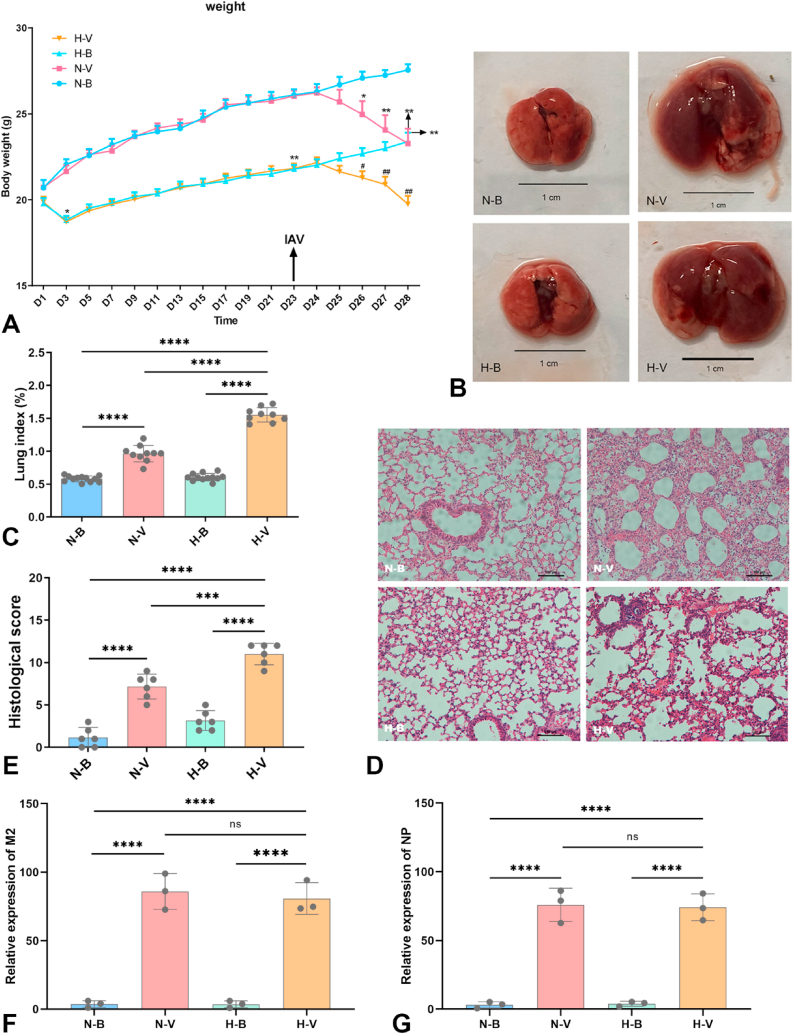
**Exposure to a humid heat environment exacerbates lung tissue injury in IAV-infected mice.** (**A**) Body weight of mice from Day 1 to Day 28, (∗*P* < 0.05, ∗∗*P* < 0.01 vs. N-B); (^#^*P* < 0.05, ^##^*P* < 0.01 vs. H-B). (**B**) Gross examination of lung tissue; scale bar = 1 cm. (**C**) Lung index. (**D**) Histopathological changes in lung tissue; scale bar = 100 μm. (**E**) Pathological score of lung tissue. (**F**) Relative expression of the viral M2 gene and (**G**) relative expression of the viral NP gene. Data are presented as mean ± SEM (**A**) or mean ± SD (**C**, **E**-**G**), ∗*P* < 0.05, ∗∗*P* < 0.01, ∗∗∗*P* < 0.001, ∗∗∗∗*P* < 0.0001. Statistical significance was evaluated using one-way ANOVA. Group abbreviations are as follows: N–B: N-B, normal environment control; N-V, normal environment with IAV infection; H-B, humid heat environment control; H-V, humid heat environment with IAV infection.

Mice in the humid heat environment group exhibited body weight loss on Days 1–3 and a gradual gain on Days 4–23 of the study. The body weights of mice in the H-B and H-V groups were significantly lower than those of the N-B group (Fig. 2A). The lung index of mice, calculated as (lung weight/body weight) %, was significantly higher in the H-V group compared to the N-V group (Fig. 2C). Gross examination and histopathological analysis revealed more severe lung tissue injury in the H-V group (Fig. 2B–D, E). However, no effect on IAV replication was observed, nor were there significant differences in the relative expression of M2 and NP genes between mice in the N-V and H-V groups (Fig. 2F and G).

### 3.2 Intestinal mucosal immunity

**Fig. 3:**
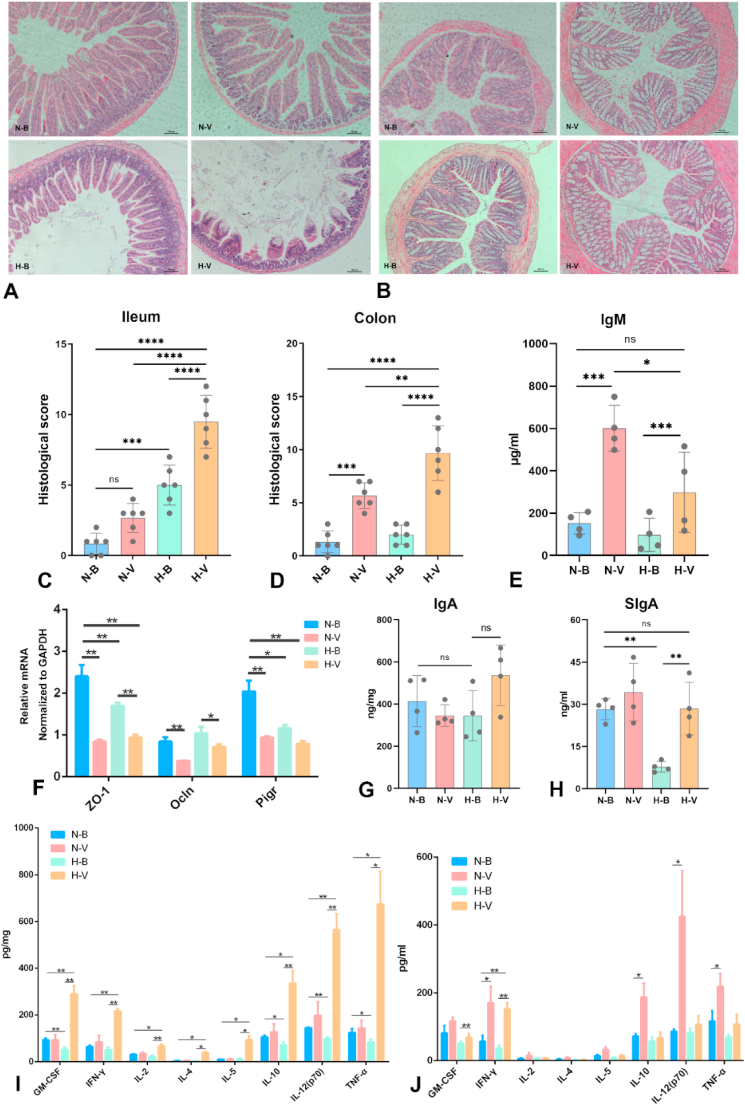
**Impact of humid heat environment exposure on intestinal mucosal immunity in IAV-infected mice**. **(A**, **B**) Pathological changes in the ileum (A) and colon (B); scale bar = 100 μm. (**C, D**) Corresponding pathological scores for the ileum (**C**) and colon (**D**). (**E**) Total plasma IgM levels. (**F**) Relative mRNA expression of tight junction proteins (*ZO-1*, *Ocln*) and the polymeric immunoglobulin receptor (*pIgR*) in colon tissue. (**G**, **H**) Levels of IgA (G) and SIgA (**H**) in the ileum. (**I**, **J**) Th1/Th2-related cytokine levels in the colon (**I**) (n = 6) and plasma (**J**) (n = 6). Data are presented as mean ± SD. ∗*P* < 0.05, ∗∗*P* < 0.01, ∗∗∗*P* < 0.001, ∗∗∗∗*P* < 0.0001. Statistical significance was evaluated using one-way ANOVA. Abbreviations: N-B, normal environment control; N-V, normal environment with IAV infection; H-B, humid heat environment control; H-V, humid heat environment with IAV infection.

IAV infection resulted in serrated changes in the small intestine, accompanied by glandular and villus loss. Mice exposed to the humid heat environment exhibited a thickening muscle layer and a reduction in the number and length of the small intestinal villi compared to the N-B group (Fig. 3A–C). For mice in the H-V group, the small intestinal villi were significantly shortened, exhibiting degenerate, necrotic, and exfoliated. Similarly, exposure to a humid heat environment also exacerbated the pathological damage to colonic tissue in IAV-infected mice. Compared to other groups, the colons of mice in the H-V group showed a significantly higher number of goblet cells and increased mucus secretion (Fig. 3B–D).

qRT‒PCR analysis revealed that the gene expression of *ZO-1* and polymeric immunoglobulin receptor (*Pigr*) was significantly higher in the blank group than in the other three groups (Fig. 3F). Gene expression of *ZO-1* in the H-V group was further downregulated compared with that in the H-B group. The mRNA levels of *Ocln* were significantly decreased in IAV-infected mice compared to uninfected controls. However, no significant differences in *Ocln* expression were detected between the N-B and H-B groups, nor were changes observed in IgA protein expression in ileum tissue among the four groups. IAV infection did not significantly alter SIgA levels, although the expression of SIgA was significantly reduced in the H-B group (Fig. 3G and H).

Serum IgM levels were significantly elevated in IAV-infected mice; however, they were significantly reduced in the H-V group compared to the N-V group (Fig. 3E). Conversely, IAV-infected mice exposed to a humid heat environment exhibited inhibition of Th1/Th2-related cytokine production in the colon. Compared to the N-B group, the levels of GM-CSF, IL-10, IL-12 (p70), and TNF-α in the H-B group were significantly decreased, accompanied by a decreasing trend in IFN-γ and IL-2 (Fig. 3I). Regarding cytokines in plasma, only IFN-γ levels in the H-V group were significantly increased compared with those in the N-B group (Fig. 3J). Furthermore, the expression of IFN-γ, IL-10, IL-12 (p70), and TNF-α in the N-V group was significantly higher than that in the N-B group.

### 3.3 16S compositional analysis of cecal microbiota

**Fig. 4:**
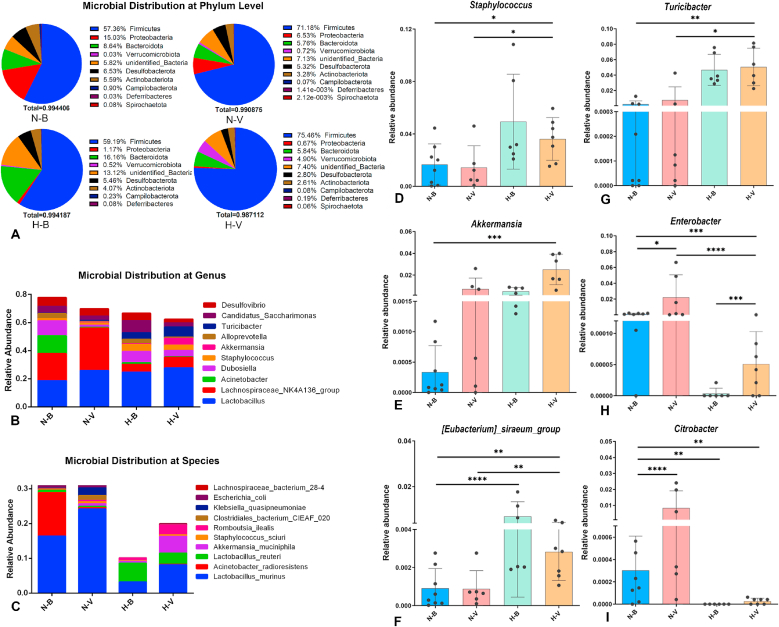
**Composition and relative abundance of the cecal microbiota.** (**A**) Relative abundance of the top 10 taxa at the phylum level. (**B**) Microbial distribution at the genus level. (**C**) Microbial distribution at the species level. (**D**–**I**) Relative abundance of *Staphylococcus* (**D**), *Akkermansia* (**E**), *Eubacterium siraeum* group (**F**), *Turicibacter* (**G**), *Enterobacter* (**H**), and *Citrobacter* (**I**). Data are presented as mean ± SD. ∗*P* < 0.05, ∗∗*P* < 0.01, ∗∗∗*P* < 0.001, ∗∗∗∗*P* < 0.0001. Statistical significance was evaluated using one-way ANOVA. Abbreviations: N-B, normal environment control; N-V, normal environment with IAV infection; H-B, humid heat environment control; H-V, humid heat environment with IAV infection.

Alpha diversity analysis revealed distinct differences in microbiota richness and diversity between the N-B group and the H-V group. The alpha diversity indices of ACE, Chao1, and PD_whole_tree in the H-V group were significantly higher than those in the N-B and H-B groups (Figs. S1A, B, E). Furthermore, there were significant differences in the Shannon and Simpson indices between the N-B and H-B groups (Fig. S1C and D). Taxonomic composition was analyzed to identify the relative abundance of cecum microbiota (Fig. 4). Analysis at the phylum level (Fig. 4A) showed that the cecum microbiota of blank murine was dominated by *Firmicutes* and *Proteobacteria*. Compared to the blank mice, *Proteobacteria* abundance was significantly decreased in IAV-infected mice. Exposure to a humid heat environment further reduced the relative abundance of *Proteobacteria* in IAV-infected mice. At the genus level, the murine cecum microbiota was dominated by *Lactobacillus*, the abundance of which was higher in the H-V group than in the blank group (Fig. 4B). Significant increases in genera *Staphylococcus and Akkermansia* genera in the H-V group were accompanied by a significant decrease in the relative abundance of *Enterobacter* and *Citrobacter* (Fig. 4D, E, H, I).

Consistent with these results, at the species level, *Lactobacillus murinus* and *Acinetobacter radioresistens* were the dominant species in blank mice (Fig. 4C). A significant increase in *L. murinus* was observed in the N-V group compared with the N-B group. Furthermore, exposure to a humid heat environment resulted in a significant increase in the abundance of *Akkermansia muciniphila*. Conversely, lower abundance of *L. murinus* was detected in the H-V group than in the N-B and N-V groups. The cumulative proportion of the top 10 bacterial species in mice exposed to the humid heat environment was significantly lower than that in mice in the normal environment.

### 3.4 Difference analysis of cecum metabolite by UPLU-MS/MS

**Fig. 5:**
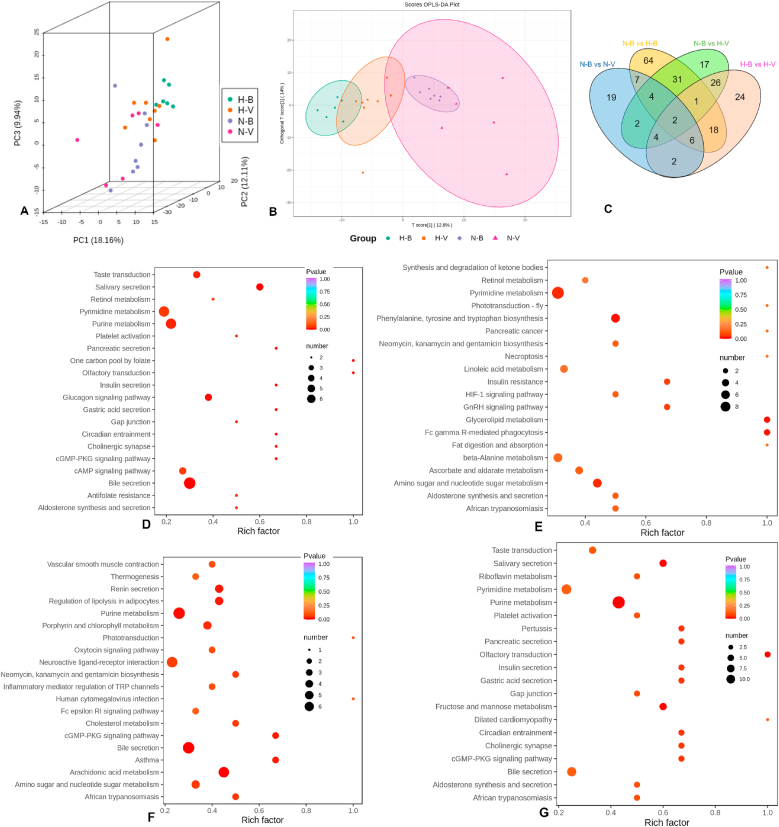
**Differential analysis of cecal metabolites.** (**A**) 3D PCA score plots of cecal metabolites from the four study groups. (**B**) OPLS-DA score plots. Group color coding: purple, N-B; red, N-V; green, H-B; orange, H-V. (**C**) Venn diagram showing the overlap of differential metabolites among comparisons. (**D**–**G**) KEGG pathway enrichment analysis of differential metabolites for the comparisons: N-B vs. N-V (**D**), N-B vs. H-B (**E**), H-B vs. H-V (**F**), and N-V vs. H-V (**G**). The rich factor represents the ratio of differential metabolites to the total number of annotated metabolites in the pathway. Higher rich factors indicate a greater degree of enrichment. P-values indicate the significance of enrichment**.** The size of the dots represents the number of differential metabolites enriched in the corresponding pathway. Abbreviations: N-B, normal environment control; N-V, normal environment with IAV infection; H-B, humid heat environment control; H-V, humid heat environment with IAV infection. (For interpretation of the references to color in this figure legend, the reader is referred to the Web version of this article.)

PCA and OPLS-DA revealed a clear separation of the cecum metabolite profiles in the blank group versus groups with humid heat environment administration (Fig. 5A and B). A Venn diagram displaying the differential metabolites (Fig. 5C) indicated that humid heat environment administration had the most significant effect on the metabolite composition of cecum contents in mice. There were 133 differential metabolites between the N-B and H-B group, compared to 46 between the N-B and N-V groups. Furthermore, 87 differential metabolites were identified between the N-B and H-V group, and 83 between the H-B and H-V group. Notably, 33 common metabolites exhibited differential abundance in both “N-B vs H-V” and “H-B vs H-V” comparisons, and 39 common metabolites were found in “N-B vs H-B” and “N-B vs H-V”. A comprehensive list of all differential metabolites is provided in Supplementary Excel.

Based on the identified of differential metabolites, KEGG pathway enrichment analysis was performed. In addition to purine and pyrimidine metabolism, the analysis of cecal differential metabolites between the N-B group and N-V group highlighted “bile secretion” and “salivary secretion” pathways (Fig. 5D). The pathways of “phenylalanine, tyrosine, and tryptophan biosynthesis”, “glycerolipid metabolism” and “Fc gamma R-mediated phagocytosis” were significantly enriched in the N-B and H-B comparison (Fig. 5E). Notably, the KEEG enrichment analysis between the H-B and H-V groups revealed the involvement of more signaling pathways than those in the comparison of other groups (Fig. 5F). Furthermore, KEGG pathway enrichment analysis of differential metabolites between the N-V and H-V groups identified “cGMP-PKG signaling” and “cholinergic synapse activity” as statistically significant pathways. (Fig. 5G).

The levels of SCFAs were quantified using high-performance gas chromatography. No significant differences were found in total SCFA levels among the four groups, nor were there significant differences in the levels of the seven individual SCFAs (Fig. S2). Although both IAV infection and exposure to a humid heat environment appeared to influence SCFA distribution (Fig. S3), these variation were not statistically significant.

### 3.5 Behavior changes associated with influenza symptoms

**Fig. 6:**
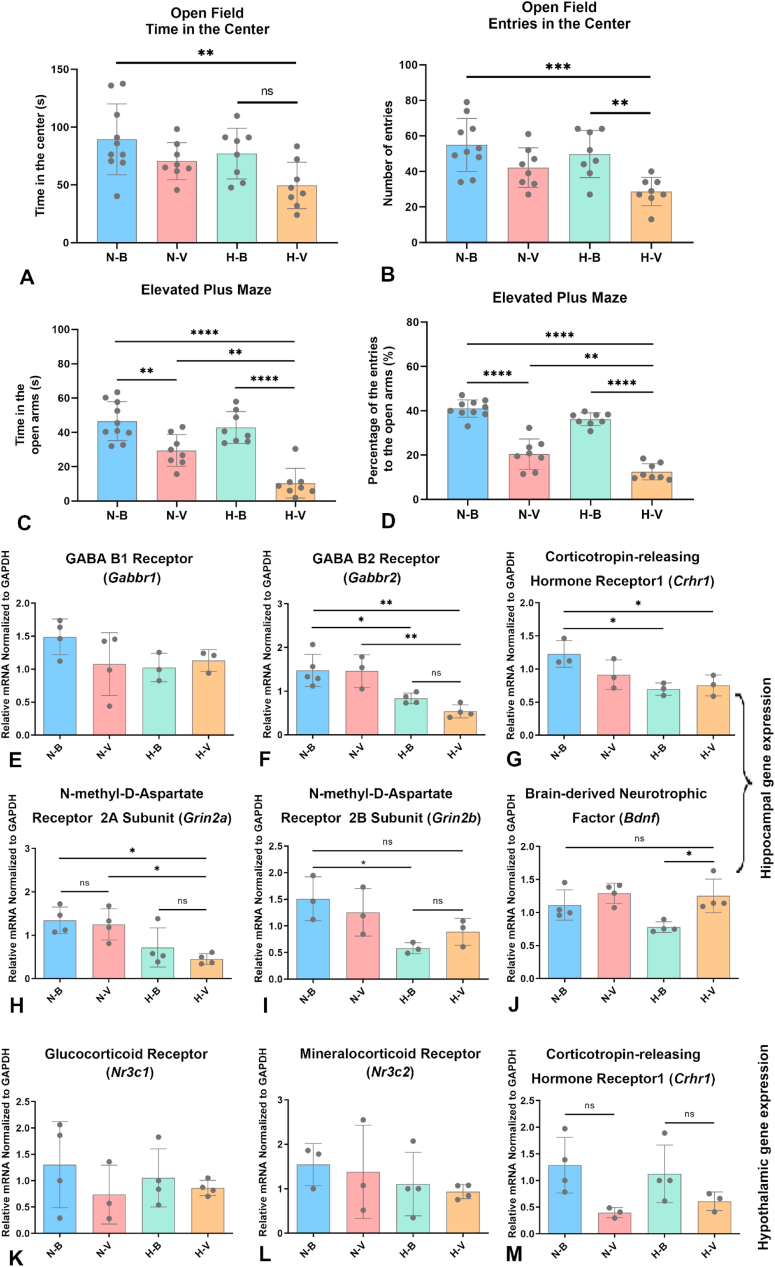
**Behavioral changes and neurotransmitter-related gene expression in IAV-infected mice.** (**A**–**D**) Behavioral assessment. (**A**) Time spent in the center and (**B**) entries into the center of the open field. (**C**) Time spent in the open arms and (**D**) percentage of entries into the open arms of the elevated plus maze. (**E**–**J**) Hippocampal gene expression. Relative mRNA levels of the GABAB1 receptor (*Gabbr1*) (**E**), GABAB2 receptor (*Gabbr2*) (**F**), corticotropin-releasing hormone receptor 1 (*Crhr1*) (**G**), NMDA receptor 2A subunit (*Grin2a*) (**H**), NMDA receptor 2B subunit (*Grin2b*) (**I**), and brain-derived neurotrophic factor (*Bdnf*) (**J**). (**K**–**M**) Hypothalamic gene expression. Relative mRNA levels of the glucocorticoid receptor (*Nr3c1*) (**K**), mineralocorticoid receptor (*Nr3c2*) (**L**), and *Crhr1* (**M**). Data are presented as mean ± SD. ∗*P* < 0.05, ∗∗*P* < 0.01, ∗∗∗*P* < 0.001, ∗∗∗∗*P* < 0.0001. Statistical significance was evaluated using one-way ANOVA. Abbreviations: N-B, normal environment control; N-V, normal environment with IAV infection; H-B, humid heat environment control; H-V, humid heat environment with IAV infection.

Due to the significantly increased mortality risk associated with tail suspension tests and forced swimming tests in this model, the OFT and EPM were employed to assess the behavioral effects of the mice in H-V group. IAV infection (N-V group) resulted in a non-significant decrease in the time spent in the center of the OFT and was associated with a tendency to make fewer entries into the center, although no significant differences were observed (Fig. 6A and B). However, mice in the H-V group exhibited a significant reduction in both the duration of time spent and the number of entries into the center. There was a trend for IAV infection to reduce the time spent in the open arms of the EPM, accompanied by a significant decrease in the percentage of entries into the open arms (Fig. 6C and D). Humid heat environment administration in IAV-infected mice (H-V) further reduced the time spent in the open arms and the number of entries into the open arms compared with the mice in the N-V group.

### 3.6 Hippocampal and hypothalamic gene expression

Compared to mice in the blank group, mice in the other groups showed moderately reduced expression of the *Gabbr1* genes, though these differences were not statistically significant (Fig. 5E–G). IAV-infected mice exposed to the humid heat environment had significantly decreased mRNA levels of the *Gabbr2* and *Grin2a* genes (Fig. 6F–H). No significant changes were observed in hippocampal *Gabbr2*, *Crhr1* or *Grin2a/b* expression between the IAV-infected group and the blank group (Fig. 6F–I); however, IAV-infected mice exposed to the humid heat environment exhibited a significant downregulation of these genes in the hippocampus compared with the blank group. Neither IAV infection nor exposure to a humid heat environment significantly altered the mRNA levels of the *Bdnf* gene (Fig. 6J). There was also no significant difference in glucocorticoid or mineralocorticoid receptor mRNA levels in the hypothalamus after IAV infection or exposure to humid heat environment (Fig. 6K and L). Furthermore, the expression of the *Crhr1* gene did not differ significantly among the four groups (Fig. 6M).

### 3.7 Cecum microbiota correlated with symptoms of severe influenza

Studies have shown that brain and gut microbiota exert a bidirectional regulatory effect, and alteration of the gut microbiota can change neuroendocrine function by regulating the gut-brain axis (Bercik et al., 2011; Bojović et al., 2020). Correlation analysis revealed a significantly positive association between the relative abundances of the genera *Prevotellaceae UCG.001*, *Lysinibacillus*, *Ralstonia*, and *Acinetobacter* and the duration of time spent in or entries into the center of the open field in tests to measure anxiety-like behavior. Conversely, a negative association was revealed between the relative abundances of the genera *Erysipelatoclostridium*, *Oscillibacter*, *Romboutsia*, *Akkermansia*, and *Turicibacter* and the time spent in or entries into the center (Fig. S4A). At the species level, the correlation with behavior measured by OFT was more significant than that with EPM testing. Relative abundances of *Sphingomonas leidyi*, *Ruminococcus sp*, *Rothia amarae*, *Histophilus somni*, *Caulobacter sp*, *Bradyrhizobium elkanii*, *Ralstonia pickettii*, *K. quasipneumoniae*, *C. bacterium CIEAF 020*, and *A. radioresistens* were positively associated with exploratory behavior, as reflected by the duration of time spent in and entries into the center of the open field (Fig. S4B).

## 4 Discussion

The subtropical monsoon climate in Asia occurs on the eastern coast of the subtropical continent, including the Guangdong Province in China, which is characterized by high temperature and high precipitation, similar to a humid heat environment (Huo et al., 2022). The annual periodicity of influenza A epidemics increases with latitude, such that provinces in China at intermediate latitudes experience dominant semiannual influenza A periodicity, with peaks in January–February and June–August (Yu et al., 2013). In this study, we report that a humid heat environment markedly exacerbates the hyperactivation of intestinal mucosal immunity and the disruption of the mucosal barrier, phenomena that may be attributable to alterations in the gut microbiota and metabolites in mice, notably, these effects were more pronounced in mice with IAV infection. In addition, we report that neurotransmitter production in mice exposed to a humid heat environment is altered, leading to changes in behavior and emotional state.

Exposure to a humid heat environment had a marked effect on inhibiting weight gain in mice and exacerbating lung tissue injury induced by IAV infection. Our data are in line with previous studies (Baindara et al., 2021; Chen et al., 2021), showing that IAV infection can affect the distribution and relative abundance of cecum microbiota in mice, and humid heat environment administration significantly exacerbates this change. Alterations in the gut microbiota could lead to an imbalance in the intestinal homeostasis, subsequently affecting nutrient absorption and body weight gain. It has been reported that probiotic microbial species in the digestive tract can influence systemic immunity and possibly viral pathogenesis and secondary infection comorbidities via the gut–lung axis (Baindara et al., 2021). In addition, the alteration of the gut microbiota may lead to further disorders of intestinal mucosal immunity. *A. muciniphila* has been shown to cause overactivation of intestinal mucosal immunity, leading to decreased expression of Muc-2 (Qian et al., 2022), which helps maintain homeostasis of gut bacteria and prevent the invasion of pathogenic bacteria (Perez-Vilar, 2007). The balance of Th1/Th2 cells helps eliminate pathogens and reduce tissue damage by activating immune cells and secreting related cytokines (Boyaka and McGhee, 2001). Therefore, the increased cytokines secreted by Th1/Th2 cells and the decreased expression of ZO-1 and Ocln may be attributed to the humid heat environment, which further leads to the compromise of the intestinal mucosal barrier. Concurrently, exposure to a humid heat environment can significantly increase the relative abundance of *L. reuteri*. As a recognized probiotic, *L. reuteri* can increase the secretion of SIgA in the intestinal lumen and reduce intestinal permeability, thereby helping to control intestinal inflammation (Holma et al., 2001). However, the SIgA levels were significantly reduced in the H-B group, accompanied by pathological damage to intestinal tissue. It is plausible that the proliferation of other bacterial species induced by humid heat environment exposure have inhibited the expression of SIgA and Pigr. The synergistic action of Pigr and IgA can maintain the balance of the gut microbiota and help avoid invasion by pathogenic bacteria (Kaetzel, 2014). These results indicate that exposure to a humid heat environment can disrupt the intestinal mucosal barrier by downregulating the expression of ZO-1, Ocln, SIgA, and Pigr, as well as by inducing the dysbiosis of the gut microbiota and imbalance of Th1/Th2 cytokines.

A multidimensional approach to the relationship between mood and weather highlighted the important effects of humidity on human behavior (Howarth and Hoffman, 1984). Similarly, in this study, the strongest effect on behavior was observed in IAV-infected mice exposed to a humid heat environment, with animals showing reductions in activity and exploration. People with IAV infection often experience fever, headache, fatigue, and slowed responsiveness-systemic symptoms related to neuroendocrine function. Interestingly, people with anxiety or depression show similar symptoms, such as fatigue, moodiness and slow reaction (Perin et al., 2022). The present study shows that exposure to a humid heat environment exacerbates anxiety and depression-like levels in IAV-infected mice, as measured in the EPM and OFT. In addition, changes in neurotransmitter-related gene transcription in the hippocampus and hypothalamus were also significant under humid heat environment intervention, particularly the downregulation of the *Gabbr2*, *Grin2a*, and *Grin2b* genes in the hippocampus. Gamma-aminobutyric acid B (GABA_B_) receptors are heterodimeric G-protein-coupled receptors known to be involved in learning and memory (Falsafi et al., 2015). Activation of GABA_B2_ subunits has previously been reported to alleviate anxiety-like behavior in cerebral ischemic mice (Lu et al., 2016); conversely, the reduced expression of *Gabbr2* may contribute to anxiety-like symptoms, such as fatigue and unresponsiveness. Interestingly, a surprising number of variants in the *Grin* genes have been found in patients with various neuropsychiatric disorders, including chronic cognitive impairment, depression, and autism (Korinek et al., 2024). This study shows that a humid heat environment can exacerbate IAV-induced neurological symptoms by decreasing *Grin* gene expression in the hippocampus. The modulation of glutamatergic synaptic transmission by N-methyl-D-aspartate (NMDA) receptors has antidepressant effects (Donello et al., 2019). Therefore, the anxious depression-like symptoms of IAV mice under humid heat environment exposure may be related to the downregulation of *Gabbr2*, *Crhr1*, *Grin2a*, and *Grin2b* in the hippocampus.

## 5 Conclusions

Patients with influenza often exhibit neurological symptoms such as fatigue and lethargy, which serve as indicators of illness severity (Dicky et al., 2014; VanWormer et al., 2014). Our study reveals, for the first time, the adverse effects of exposure to a humid heat environment on IAV-infected mice, effects that are likely regulated by intestinal mucosal immunity and neuroendocrine activity via the gut-brain axis. Mechanistically, studies demonstrate that a humid heat environment induces the gut microbiota and metabolic dysbiosis, which may further exacerbate intestinal mucosal barrier injury. Moreover, we show that altered neurotransmitter secretion associated with humid heat environment exposure mediates the interplay between gut microbiota, metabolic dysbiosis, and IAV infection-induced anxiety- and depression-like behaviors. This study provides new theoretical insights and potential targets for managing IAV infection, which may contribute to a better understanding of the effects of humid heat environment exposure on IAV through the gut-brain axis and neuroendocrine system. The influence of environment factors on influenza pathology and brain-gut axis is complex; therefore, more in-depth studies are warranted to fully elucidate the underlying mechanisms. Therefore, when treating influenza in clinical practice, we can adopt a more comprehensive and efficient approach.

## CRediT authorship contribution statement

**Sizhi Wu:** Writing – review & editing, Writing – original draft, Validation, Resources, Methodology, Investigation, Funding acquisition, Formal analysis. **Yiwen Lv:** Writing – review & editing, Validation, Investigation. **Peng Pang:** Writing – review & editing, Funding acquisition. **Huachong Xu:** Investigation, Formal analysis. **Li Deng:** Writing – review & editing, Resources, Funding acquisition. **Wei Ma:** Supervision, Funding acquisition, Conceptualization. **Xiaoyin Chen:** Supervision, Resources, Funding acquisition, Conceptualization.

## Ethical statement

This study did not involve any human samples and animals used in this study was approved by the Animal Care and Use Committee of Jinan University. The ethics approval number is 20160616103002.

## Funding statement

This study was supported by the National Natural Science Foundation of China [grants numbers, 82204811, 82204992, 82474370, and 82374319]; and the Guangzhou Municipal Science and Technology Project [grants numbers 2023A04J0628, 202206010099]. We acknowledge and appreciate our colleagues for their valuable input and comments on this paper.

## Declaration of competing interest

The authors declare that they have no known competing financial interests or personal relationships that could have appeared to influence the work reported in this paper.

## Data Availability

Data will be made available on request.

## Abbreviations

IAV: influenza A virus; SPF: Specific Pathogen Free; N-B: a normal environment exposure for blank; N-V: a normal environment exposure with influenza A virus infection; H-B: a humid heat environment exposure; H-V: a humid heat environment exposure with influenza A virus infection group; qRT-PCR: quantitative time-polymerase chain reaction; OFT: open field test; EPM: elevated plus maze; SIgA: secretory immunoglobulin A; PCA: principal component analysis; OPLS-DA: orthogonal partial least squares-discriminant analysis; KEGG: Kyoto Encyclopedia of Genes and Genomes; PIGR: polymeric immunoglobulin receptor; GABA: Gamma-aminobutyric acid; NMDA: N-methyl-D-aspartate.
