# Supplementary material for: Distinct actions of the humid heat environment on host gut microbiota, intestinal mucosal immunity, neuroendocrinology in influenza A virus-infected mouse
Source: Brain Behav Immun Health. 2025 Dec 19;51:101164. doi: 10.1016/j.bbih.2025.101164 (PMC12813360; doi:10.1016/j.bbih.2025.101164)
Supplement: Multimedia component 1 [file mmc1.docx]

**Distinct actions of the** **humid heat environment on host** **gut microbiota,** **intestinal mucosal immunity****, neuroendocrinology in** **influenza A virus-infected mouse**

1. **Supplemental Methods and Materials**
   1. **Plasma and Tissue Collection**

At the end of the experiment, blood was collected from each mouse via retro-orbital bleeding into 1.5 mL centrifuge tubes containing heparin anticoagulant. The blood was mixed with heparin by gently inverting the tube and centrifuged at 3500 × g at 4 °C for 15 minutes. Plasma was carefully aspirated and stored at -80 °C. The mice were then euthanized by cervical dislocation. The brain was quickly excised, and the hippocampus and hypothalamus were dissected, snap-frozen on dry ice, and stored at -80 °C for further neurotransmitter analysis. Lung tissue was then excised and stored at -80 °C for detection of lung infection. Subsequently, the intestinal tissue was excised and stored at -80 °C for further analysis, and the cecal contents were snap-frozen on dry ice and then stored at -80 °C for further analysis of microbiota, short-chain fatty acids (SCFAs), and metabolites.

- 1. **Histopathology Examination**

Lung and intestinal tissue samples were fixed in 4% paraformaldehyde, dehydrated in graded ethanol, cleared, and embedded in paraffin. The tissues were sectioned at a thickness of 5 μm using a microtome. The tissue sections were dewaxed with xylene and rehydrated with graded ethanol, followed by Hematoxylin and Eosin (H&E) staining. Stained sections were dehydrated with graded alcohol, cleared, and sealed with neutral resin. Images were captured using an optical microscope (OLYMPUS, Japan) equipped with a camera (Nikon, ECLIPSE 80i, Japan). Lung pathology was scored based on epithelial tissue damage, hemorrhagic congestion, mucosal edema, and neutrophil infiltration. For colon histopathologic scoring, the evaluated parameters were mucosal thickening, goblet cell depletion, cellular infiltration, and tissue damage. In addition, the length and number of villi were evaluated **f**or the small intestinal histological score. Each parameter was scored on a scale from 0 (normal) to 3 (severe change). The overall histological score was calculated as the sum of the individual parameter scores.

**1.3 Quantitative Real-Time Polymerase Chain Reaction (qRT-PCR)**

Total RNA was extracted from lung tissues using RNAiso Plus (Takara, Japan) according to the manufacturer’s instructions. RNA was reverse transcribed into cDNA using the PrimeScript™ RT Reagent Kit with gDNA Eraser (Takara, Japan). The reverse transcription reaction was incubated at 37 °C for 15 min and 85 °C for 5 s, then held at 4 °C. Real-time PCR was performed using SYBR Premix Ex Taq II (Takara, Japan) on a CFX Connect Real-Time PCR Detection System (BIO-RAD, USA). The thermal cycling conditions were initial denaturation (optional, e.g., 95 °C for 30 s), followed by 39 cycles of 95 °C for 5 s and 60 °C for 30 s. Primers were synthesized by Sangon Biotech (Shanghai, China) and are listed in Supplementary Table S1. Relative mRNA expression levels were calculated using the 2−ΔΔCt method, with *Gapdh* as the internal control.

**1.4 Enzyme Linked Immunosorbent Assay** **(Elisa)**

Plasma IgM levels and ileal IgA and SIgA levels were quantified using ELISA kits (MultiSciences, China). All reagents and samples were equilibrated to room temperature and diluted according to the manufacturer's instructions. Standards and samples were added to the plates, which were then sealed and incubated with shaking (300 rpm) at room temperature for 2 hours using a microplate shaker (Thermo Fisher, MB100-2A, USA). The plates were washed at least six times. Subsequently, the detection antibody was added to each well, and the plates were sealed and incubated with shaking (300 rpm) at room temperature for 1 hour. Following incubation with TMB substrate in the dark, the reaction was terminated by adding the stop solution. Absorbance was measured using a multimode microplate reader (Varioskan LUX, Thermo Fisher, USA), and the concentrations of IgM, IgA, or SIgA were calculated based on the standard curves.

**1.5** **Cytokine Quantification using** **Bio-Plex Multiplex Assay**

Lung and intestinal tissues were homogenized, and protein concentrations were measured using an Enhanced BCA Protein Assay Kit (Beyotime, Shanghai, China). Cytokine levels were determined using a Bio-Plex Pro™ Cytokine Reagent Kit (Bio-Rad, USA). Tissue homogenates and plasma samples were diluted based on protein concentration, and standards were serially diluted. Standards and samples were added to the corresponding wells of the reaction plate, followed by the addition of magnetic beads. The plate was **sealed** and incubated with shaking at 850 rpm at room temperature for 30 minutes. After washing the plate three times using a Bio-Plex magnetic separator, detection antibody was added for incubation. Subsequently, the plate was washed, and Streptavidin-PE was added for further incubation. Finally, the beads were resuspended in Assay Buffer. Data acquisition was performed on a Bio-Plex system according to the manufacturer's instructions, and data were analyzed using Bio-Plex Manager 6.1 software.

**1.6** **16S rRNA Gene Sequencing and Analysis**

Total genomic DNA was extracted from cecal contents using the GenElute™ Fecal DNA Isolation Kit (Sigma-Aldrich, Germany). DNA integrity and concentration were assessed by 1% agarose gel electrophoresis. The hypervariable V3-V4 region of the bacterial 16S rRNA gene was amplified using the primers 338F (5’-ACTCCTACGGGAGGCAGCAG-3’) and 806R (5’-GGACTACHVGGGTWTCTAAT-3’). PCR was performed using Phusion® High-Fidelity PCR Master Mix with GC Buffer (New England Biolabs). PCR products were examined by 2% agarose gel electrophoresis and purified using a Gel Extraction Kit (QIAGEN, Germany). Sequencing libraries were generated using the TruSeq® DNA PCR-Free Sample Preparation Kit and quantified by Qubit and Q-PCR. The libraries were sequenced on an Illumina NovaSeq 6000 platform. Raw tags were filtered using QIIME (Version 1.9.1) (Caporaso et al., 2010). to obtain high-quality clean tags. Chimera sequences were detected and removed using VSEARCH (Rognes, Flouri, Nichols, Quince, & Mahé, 2016). OTU clustering and species annotation were performed using UPARSE (v7.0.1001) (Haas et al., 2011), based on the SILVA138 SSU rRNA database (Edgar, 2013). Phylogenetic relationships were analyzed using MUSCLE (Wang, Garrity, Tiedje, & Cole, 2007). Alpha and Beta diversity analyses were performed using QIIME. Alpha diversity indices, including ACE, Chao1, Shannon, Simpson, PD_whole_tree, and Good’s coverage, were calculated to evaluate species richness and diversity.

**1.7 Open Field Test**

To assess locomotor activity, exploratory behavior, and anxiety-like levels in a novel environment, mice were first exposed to 95 dB of white noise for 1 hour (stress induction). Subsequently, they were placed into an open arena (40 × 32 × 23 cm) under 60 lux lighting. The activity of the mice was recorded for 5 minutes, including horizontal movement, rearing counts (vertical movement), and defecation (fecal boli). Between trials, the arena was thoroughly cleaned with 70% ethanol solution to eliminate olfactory cues and dried with paper towels. Experiments were videotaped using a ceiling-mounted camera, and parameters were analyzed using EthoVision XT software (Version 14.0, Noldus, UK).

**1.8 Elevated plus maze**

Anxiety-like behavior was assessed based on the conflict between the animal's innate exploratory drive and its aversion to open, elevated spaces. The apparatus consisted of a gray plastic cross-shaped maze elevated 1 m above the floor, comprising two open arms and two closed arms (50 × 5 cm). The closed arms were enclosed by 15 cm high walls, while the open arms had 1 cm high ledges. Mice were individually placed in the center of the maze facing an open arm and allowed 5 minutes of free exploration. The number of entries into and the time spent in the open and closed arms were recorded**.** The percentages of time spent in the open arms and open arm entries were calculated. Experiments were videotaped using a ceiling-mounted camera and analyzed using EthoVision XT software (Version 14.0, Noldus, UK).

**1.9 Ultra-performance Liquid Chromatography/Tandem Mass Spectrometry (UPLC-MS/MS) Analysis**

Cecal contents were thawed on ice. Samples (50 mg) were homogenized with 500 μL of ice-cold methanol/water (70%, v/v) containing an internal standard (MetWare, China). The samples were vortexed for 3 min, sonicated for 10 min in an ice-water bath, and vortexed again for 1 min. After centrifugation at 12,000 rpm for 10 min at 4 °C, 250 μL of the supernatant was collected and centrifuged again at 12,000 rpm for 5 min at 4 °C. Subsequently, 150 μL of the supernatant was used for analysis via Ultra-Performance Liquid Chromatography (UPLC, ExionLC™ AD, SCIEX) and Tandem Mass Spectrometry (MS/MS, QTRAP**®**, SCIEX). Analytical conditions were set according to the manufacturer's recommendations. Linear ion trap (LIT) and triple quadrupole (QQQ) scans were acquired on a triple quadrupole-linear ion trap mass spectrometer (QTRAP® LC-MS/MS System) equipped with an ESI Turbo Ion-Spray interface, operating in positive and negative ion modes and controlled by Analyst 1.6.3 software (SCIEX) (Fraga, Clowers, Moore, & Zink, 2010). A specific set of MRM transitions were monitored for each period according to the metabolites eluted (Eriksson, Kettanehwold, Trygg, Wikström, & Wold, 2006). Differential metabolites between groups were determined based on variable importance in projection (VIP) ≥ 1, |Log2FC| ≥ 1.0 and *P*-value < 0.05 (Thévenot, Roux, Xu, Ezan, & Junot, 2015). To avoid overfitting, a permutation test was performed. Identified metabolites were annotated using the KEGG Compound database, and annotated metabolites were then mapped to the KEGG Pathway database.

**1.10** **Quantification of SCFAs**

SCFA extraction was performed following standard protocols (MetWare Biotechnology, Wuhan, China). Briefly, 20 mg of fecal samples were homogenized in phosphoric acid solution (0.5% v/v), vortexed, and ultrasonicated. After centrifugation (12,000 rpm, 10 min, 4°C), the supernatant was extracted with methyl tert-butyl ether (MTBE) containing internal standards. The mixture was vortexed, ultrasonicated, and centrifuged again. The final supernatant was collected for GC-MS/MS analysis. Analysis was performed using an Agilent 7890B gas chromatograph coupled to a 7000D mass spectrometer equipped with a DB-5MS column (30 m × 0.25 mm × 0.25 μm; J&W Scientific, USA). Helium was used as the carrier gas at a flow rate of 1.2 mL/min. Injection was performed in splitless mode (2 μL volume) with injector and transfer line temperatures set at 200°C and 230°C, respectively. The oven temperature program was initiated at 90°C (held for 1 min), increased to 100°C (25°C/min), then to 150°C (20°C/min, held for 0.6 min), and finally to 200°C (25°C/min, held for 0.5 min). Data acquisition was performed in multiple reaction monitoring (MRM) mode.

**2 Supplemental figures and tables**


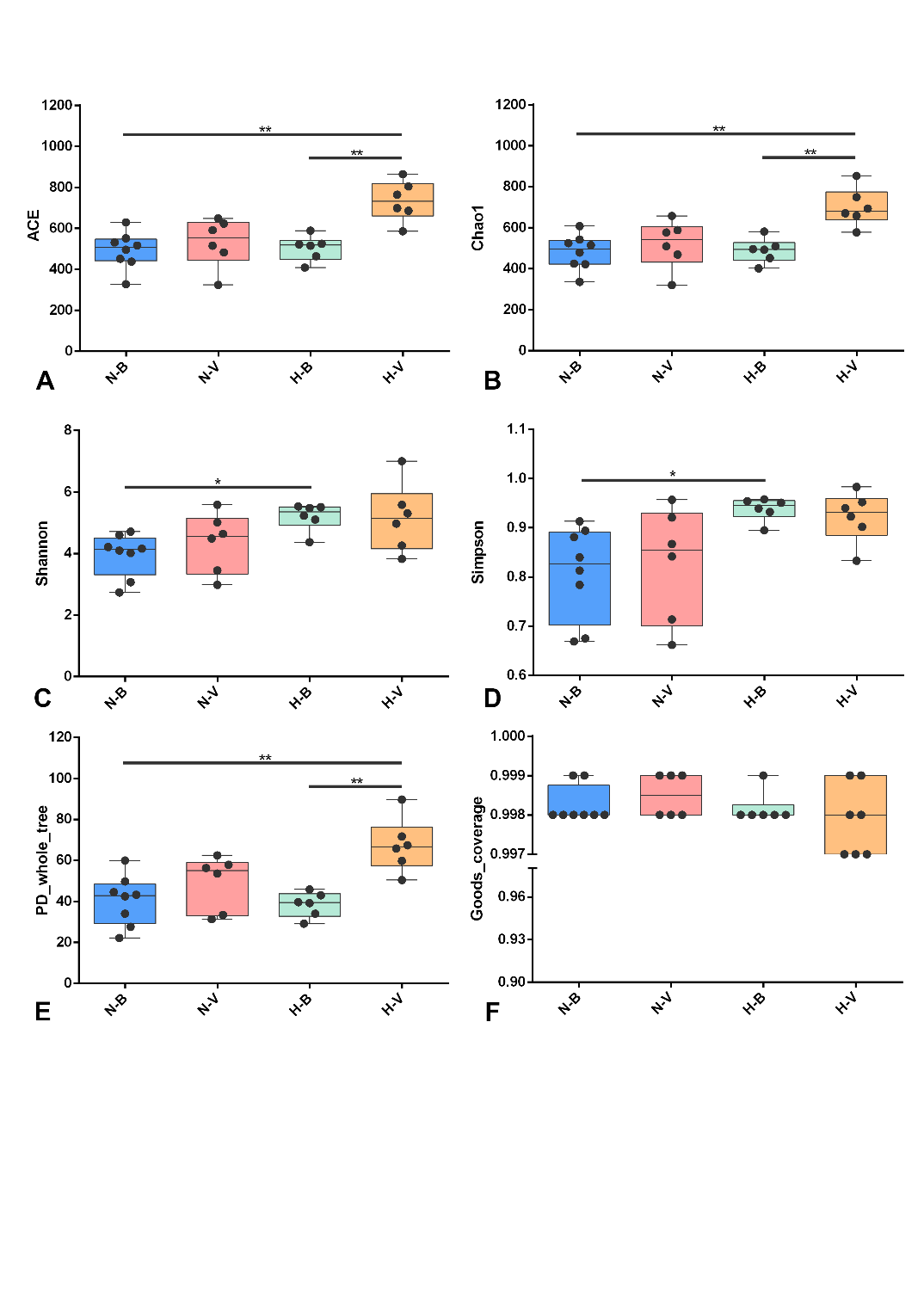


**Fig S1. Alpha diversity indices of the cecal microbiota.** ACE (**A**), Chao1 (**B**), Shannon (**C**), Simpson (**D**), PD_whole_tree (**E**), Goods_coverage (**F**). (A) ACE index. (B) Chao1 index. (C) Shannon index. (D) Simpson index. (E) PD_whole_tree index. (F) Good’s coverage. Data are presented as boxplots. **P* < 0.05, ***P* < 0.01. Statistical significance was evaluated using one-way ANOVA. Abbreviations: N-B, normal environment control; N-V, normal environment with IAV infection; H-B, humid heat environment control; H-V, humid heat environment with IAV infection.


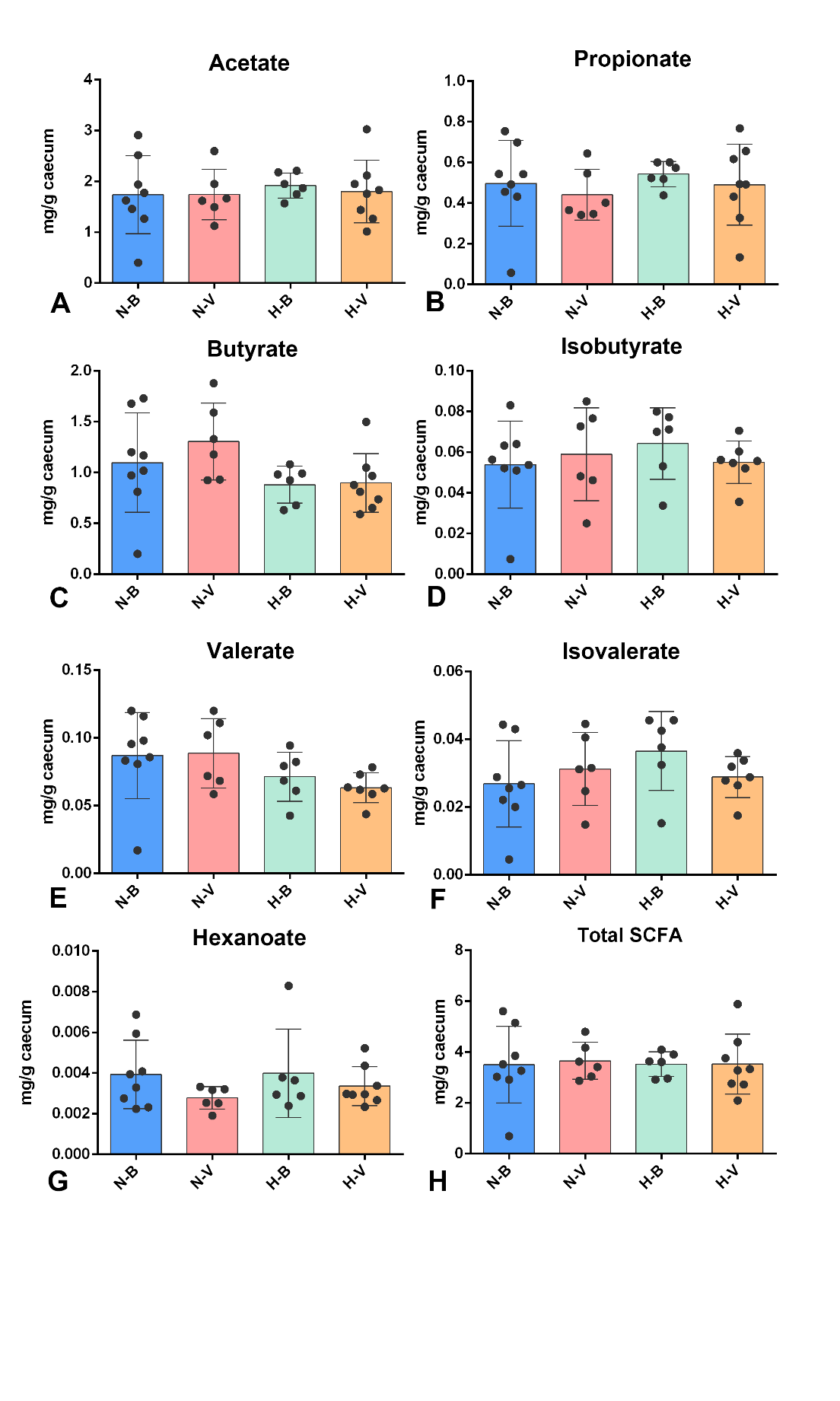


**Fig S2.** SCFA levels in cecum. Acetate (AA), Butyrate (BA), Hexanoate (HA), Isobutyrate (IBA), Isovalerate (IVA), Propionate (PA), Valerate (VA). Data are shown as the mean±SD. The significance of differences between the groups was evaluated using one-way ANOVA, and the differences were considered statistically significant at *P* < 0.05. N-B: a blank with normal environment exposure group, N-V: a normal environment exposure with IAV infection group, H-B: a humid heat environment exposure group, H-V: and a humid heat environment exposure with IAV infection group.

**
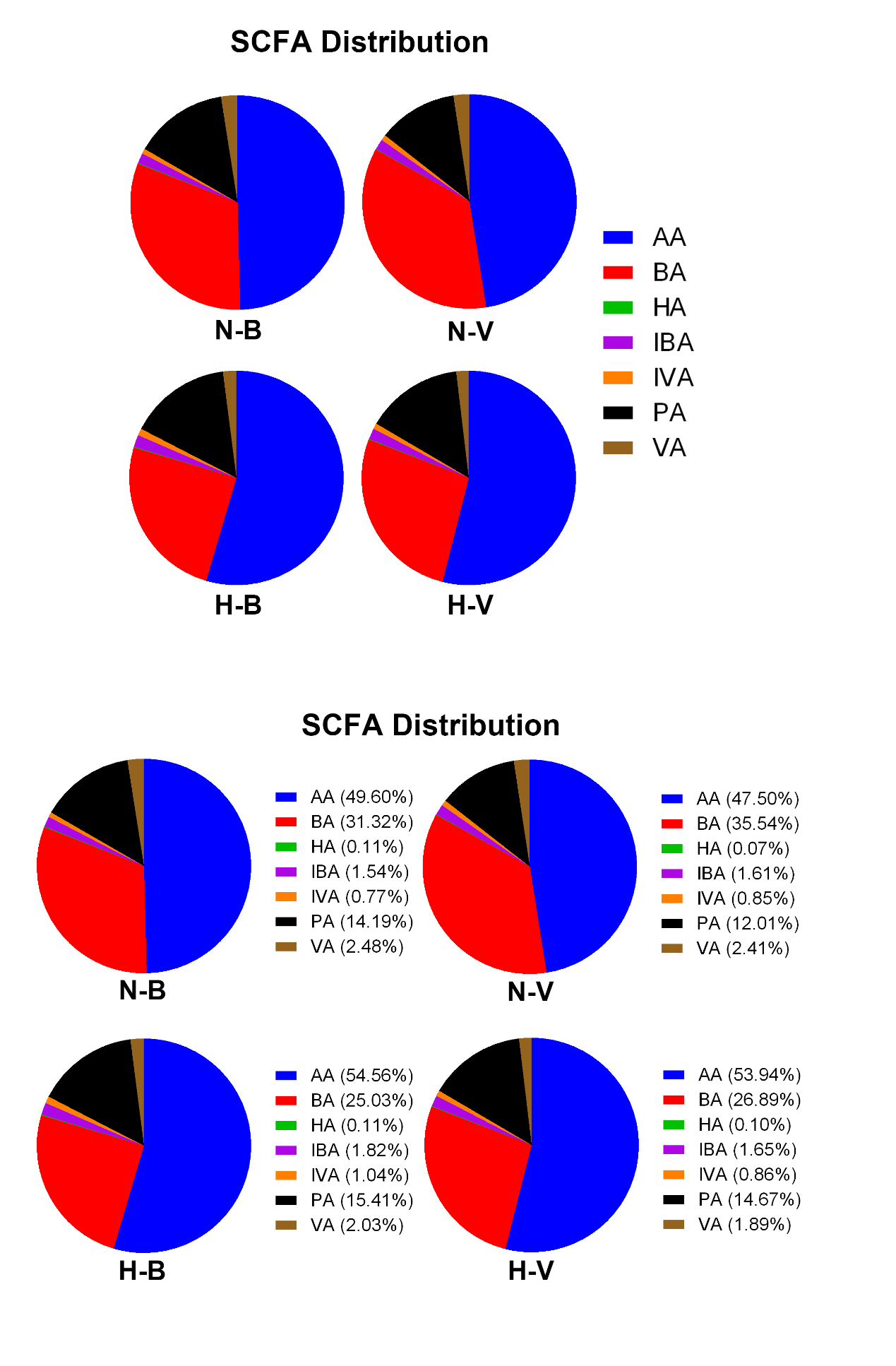
**

**Fig S3.** **Concentrations of short-chain fatty acids (SCFAs) in cecal contents.**
Levels of individual SCFAs: Acetate (AA), Butyrate (BA), Hexanoate (HA), Isobutyrate (IBA), Isovalerate (IVA), Propionate (PA), and Valerate (VA). Data are presented as mean ± SD. Statistical significance was evaluated using one-way ANOVA. Abbreviations: N-B, normal environment control; N-V, normal environment with IAV infection; H-B, humid heat environment control; H-V, humid heat environment with IAV infection.
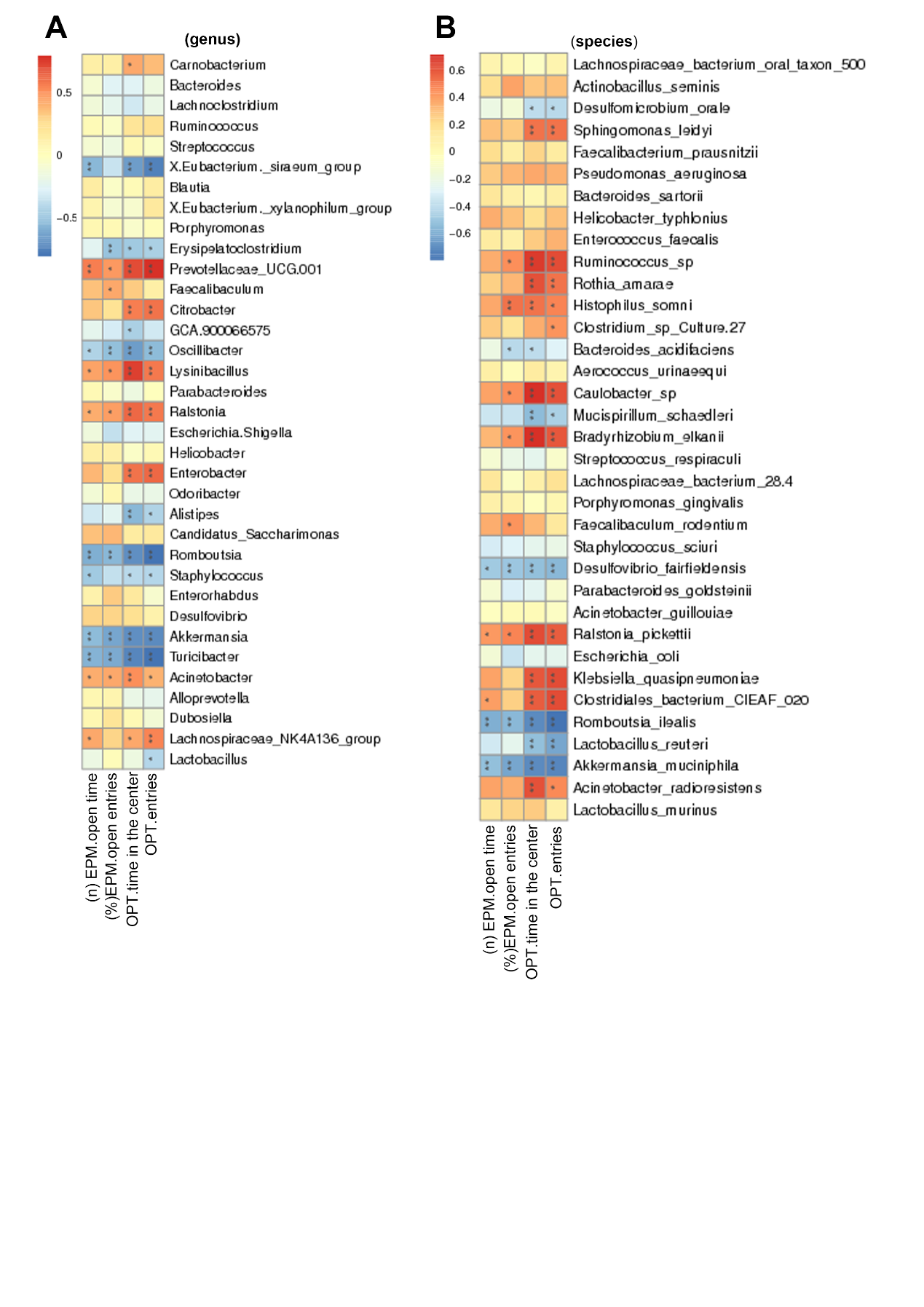


**Fig S4.** Correlation analysis between the gut microbiota and anxiety-like behaviors.
Heatmap showing Spearman’s rank correlations. The color intensity and size of the circles represent the strength of the correlation; red indicates a positive correlation, and blue indicates a negative correlation. *P < 0.05, **P < 0.01. Abbreviations: EPM, elevated plus maze test; OFT, open field test.

**Table S1.** The primers for qRT-PCR

| Gene name | Primers (5’-3’) |
| --- | --- |
| corticotropin-releasing hormone receptor 1 (Crhr1) | Forward: CATCATCCACTGGAACCTCATC |
|  | Reverse: ATCCAGAAGAAGTTGGTTACGT |
| brain-derived neurotrophic factor (Bdnf) | Forward: CCCATGAAAGAAGTAAACGTCC |
|  | Reverse: CCTTATGGTTTTCTTCGTTGGG |
| NMDA receptor 2A subunit (Grin2a) | Forward: GAACGCGAACTTCGAAATCTG |
|  | Reverse: GTCAGTGCGGTTCATCAATAAC |
| NMDA receptor 2B subunit (Grin2b) | Forward: AAGAAGAATCGGAACAAACTGC |
|  | Reverse: CAGCTGGCATCTCAAACATATG |
| GABA_B1_ receptor (Gabbr1) | Forward: CATCAAGACATTCCGTTTCCTG |
|  | Reverse: GACAGACAACAGCAAGAACAAT |
| GABA_B2_ receptor (Gabbr2) | Forward: TGTCGTTGCTGCTGTCGTTGG |
|  | Reverse: GTGACTCGTTGCGGATCTGCTC |
| glucocorticoid receptor (Nr3c1) | Forward: ACTCCAAAGAATCCTTAGCTCC |
|  | Reverse: TATACAAGTCCATCACGCTTCC |
| mineralocorticoid receptor (Nr3c2) | Forward: CCTTCAGTTCGTGCGGCTTCAG |
|  | Reverse: AGAACCTCTGCCAACTCTGTCCAG |
| Zonula Occludins protein-1(ZO-1) | Forward: GCGAACAGAAGGAGCGAGAAGAG |
|  | Reverse: GCTTTGCGGGCTGACTGGAG |
| occludin (Ocln) | Forward: TGGCTATGGAGGCGGCTATGG |
|  | Reverse: AAGGAAGCGATGAAGCAGAAGGC |
| polyimmunoglobulin receptor (Pigr) | Forward: GTAGAGTCAATGACAAGTTCGC |
|  | Reverse: AAGGACATAGGCAGAAATGTGA |
| Glyceraldehyde-3-phosphate Dehydrogenase (GAPDH) | Forward: TGATGACATCAAGAAGGTGGTGAAG |
|  | Reverse: TCCTTGGAGGCCATGTAGGCCAT |
| Nucleocapsid Protein (NP) | Forward: TGCTTCAAAACAGCCAAGTG |
|  | Reverse: GATGCCCTCTGTTGATTGGT |
| M2 | Forward: GACCGATCCTGTCACCTCTGAC |
|  | Reverse: AGGGCATTCTGGACAAAGCGTCTA |

**Excel S1.** Comparison of differential metabolites

Formula: Molecular formula. Compounds: Metabolite name. Class I: Primary chemical classification. Class II: Secondary chemical classification. CAS: CAS registry number. A: Identification confirmed by matching Q1 (precursor ion), RT (retention time), DP (declustering potential), CE (collision energy), and **two** fragment ions with the reference database. B: Identification confirmed by matching Q1, RT, DP, CE, and **one** fragment ion with the reference database.

**References**

Caporaso, J. G., Kuczynski, J., Stombaugh, J., Bittinger, K., Bushman, F. D., Costello, E. K., . . . Knight, R. (2010). QIIME allows analysis of high-throughput community sequencing data. *NATURE METHODS, 7*(5), 335-336.

Edgar, R. C. (2013). UPARSE: highly accurate OTU sequences from microbial amplicon reads. *NATURE METHODS, 10*(10), 996-998.

Eriksson, L., Kettanehwold, N., Trygg, J., Wikström, C., & Wold, S. (2006). Multi- and megavariate data analysis : Part I: Basic principles and applications. *Umetrics Inc*.

Fraga, C. G., Clowers, B. H., Moore, R. J., & Zink, E. M. (2010). Signature-discovery approach for sample matching of a nerve-agent precursor using liquid chromatography-mass spectrometry, XCMS, and chemometrics. *ANALYTICAL CHEMISTRY, 82*(10), 4165-4173.

Haas, B. J., Gevers, D., Earl, A. M., Feldgarden, M., Ward, D. V., Giannoukos, G., . . . Birren, B. W. (2011). Chimeric 16S rRNA sequence formation and detection in Sanger and 454-pyrosequenced PCR amplicons. *GENOME RESEARCH, 21*(3), 494-504.

Rognes, T., Flouri, T., Nichols, B., Quince, C., & Mahé, F. (2016). VSEARCH: a versatile open source tool for metagenomics. *PeerJ, 4*, e2584.

Thévenot, E. A., Roux, A., Xu, Y., Ezan, E., & Junot, C. (2015). Analysis of the human adult urinary metabolome variations with age, body mass index, and gender by implementing a comprehensive workflow for univariate and OPLS statistical analyses. *JOURNAL OF PROTEOME RESEARCH, 14*(8), 3322-3335.

Wang, Q., Garrity, G. M., Tiedje, J. M., & Cole, J. R. (2007). Naive Bayesian classifier for rapid assignment of rRNA sequences into the new bacterial taxonomy. *APPLIED AND ENVIRONMENTAL MICROBIOLOGY, 73*(16), 5261-5267.
